# Solitary giant neurofibroma of the neck subjected to photodynamic therapy: case study

**DOI:** 10.1186/1758-3284-4-30

**Published:** 2012-06-06

**Authors:** Zaid Hamdoon, Waseem Jerjes, Raed Al-Delayme, Colin Hopper

**Affiliations:** 1 Department of Oral and Maxillofacial Surgery, School of Dentistry, Al-Yarmouk University College, Baghdad, Iraq; 2 Unit of Oral and Maxillofacial Surgery, UCL Eastman Dental Institute, London, UK; 3 Department of Oral and Maxillofacial Surgery, University of Mosul, Mosul, Iraq; 4 Department of Surgery, UCL Medical School, London, UK; 5 Leeds Institute of Molecular Medicine, University of Leeds, Leeds, UK; 6 Department of Surgery, AL-Yarmouk University College, Baghdad, Iraq; 7 UCLH Head and Neck Centre, Euston Street, London, United Kingdom

## Abstract

Photodynamic therapy (PDT) - the fourth modality - has been successfully used in the management of early and advanced pathologies of the head and neck. We studied the effect of this modality on a giant solitary neurofibroma of the neck. A 70-year-old Caucasian female presented with left neck pain and disfigurement associated with slight shortness of breath and dysphagia. Examination revealed a large mass in the neck with no neurovascular compromise. Magnetic resonance imaging (MRI) reported a heterogeneously enhancing mass extending from the left angle of the mandible to the base of the neck. A core biopsy was performed and histopathological examination revealed a disorganised array of peripheral nerve fascicles. The patient elected to receive photodynamic therapy as the primary intervention. The multi-disciplinary meeting approved the treatment plan. The photosensitizing agent was mTHPC (0.15 mg/kg), which was systemically administered 96-hours prior to ultrasound (US)-guided light delivery to the mass, which was undertaken under general anaesthesia. Recovery was uneventful.Post-PDT follow-up showed that the patient’s pain, dysphagia and shortness of breath issues had improved. The disfigurement of the neck caused by the mass was no longer a problem. Three months post-PDT, MRI revealed a significant reduction in the neurofibroma size. PDT was proven as a successful primary intervention for this pathology. However, higher evidence-based studies are required before this therapy can be proposed as a replacement to any of the other conventional therapies.

## Introduction

Neurofibroma is a rare benign tumour arising from nerves that is composed of Schwann cells, perineural cells and fibroblasts. Neurofibromas may present either as solitary lesions or as part of a syndrome, i.e. neurofibromatosis or von Recklinghausen's disease of the skin (NF-1) [1-4].

The aetiology behind solitary neurofibroma is still unknown. Marocchio et al. [1] considered solitary neurofibroma to be hyperplastic hamartomatous malformations rather than neoplastic. Anatomically, neck neurofibromas are relatively rare, with slow painless growth. Neurofibromas are usually treated by excision, especially when they are symptomatic (i.e. pain causing swallowing or breathing problems) or disfiguring.

Photodynamic therapy (PDT) has been applied in the management of superficial and deep pathologies involving the head and neck [5-11]. PDT has been successful in managing superficial diseases such as oral premalignant disorders and early (T1/T2) oral cancer [12-14]. Successful applications were also reported in the management of nasopharyngeal carcinoma [15,16], tongue base carcinoma [17-19], subglottic carcinoma [20], vascular anomalies [5,10,21-23], sarcomas [5,10,24], pericarotid disease [25], metastatic disease to the orofacial region [26], mantle lymphoma [27] and Kimura disease [28]. The advancement in photodynamic applications suggests that PDT may be more effective when combined with other modalities, (i.e. photochemical internalization [29-33]. Optical diagnostics (i.e. optical coherence tomography) can be used to guide PDT therapy and assess outcome especially when it comes to superficial tissue disease, (i.e. skin cancer) [34].

In this case study, we describe an unusual case of solitary neurofibroma of the neck without the stigmas of NF-1, which was treated with US-guided interstitial PDT.

## Case study

A 70-year-old Caucasian female presented to the UCLH Head and Neck Unit, London, with a large left neck mass. Pain and disfigurement were the major complaints. The pain itself was described as sharp, radiating to the right ear and exacerbated by turning the head to the right. The mass had been increasing in size over the last year and causing significant disfigurement to the left side of the neck. Other complaints included slight shortness of breath, mainly on exertion, and mild dysphagia. The patient's medical history included congestive heart failure.

Clinical examination revealed a large mass of the left neck, which was slightly mobile but fixed to deeper tissue with no ulceration of the overlying skin and no transillumination. There was no neurovascular compromise and examination of the cranial nerves in the area was unremarkable.

Magnetic resonance imaging (MRI) reported a 12 × 12 × 5 cm heterogeneously enhancing mass in the left base of neck, extending from the left angle of the mandible to the base of neck. There was invasion of the surrounding structures, including the sternocleidomastoid muscle. However, there was no invasion of neighbouring vascular structures. A surgical core biopsy was performed under general anaesthesia. Histopathological assessment revealed a submucosal nodular proliferation that consisted of a disorganised array of peripheral nerve fascicles. Within the fascicles, individual nerve fibres were splayed apart by an increased endoneural matrix.

Surgical excision was initially offered as it represents the gold-standard treatment, but the patient requested an alternative less-invasive therapy. Following the multi-disciplinary meeting (MDM), the patient was offered US-guided photodynamic therapy using mTHPC as the photosensitizing agent.

The patient was sensitized 96-hours prior to light delivery by the photosensitive agent mTHPC 0.15 mg/kg. The agent was administered intravenously and the patient instructed to remain in a dimly lit room until the day of the procedure (light delivery).

**Figure 1:**
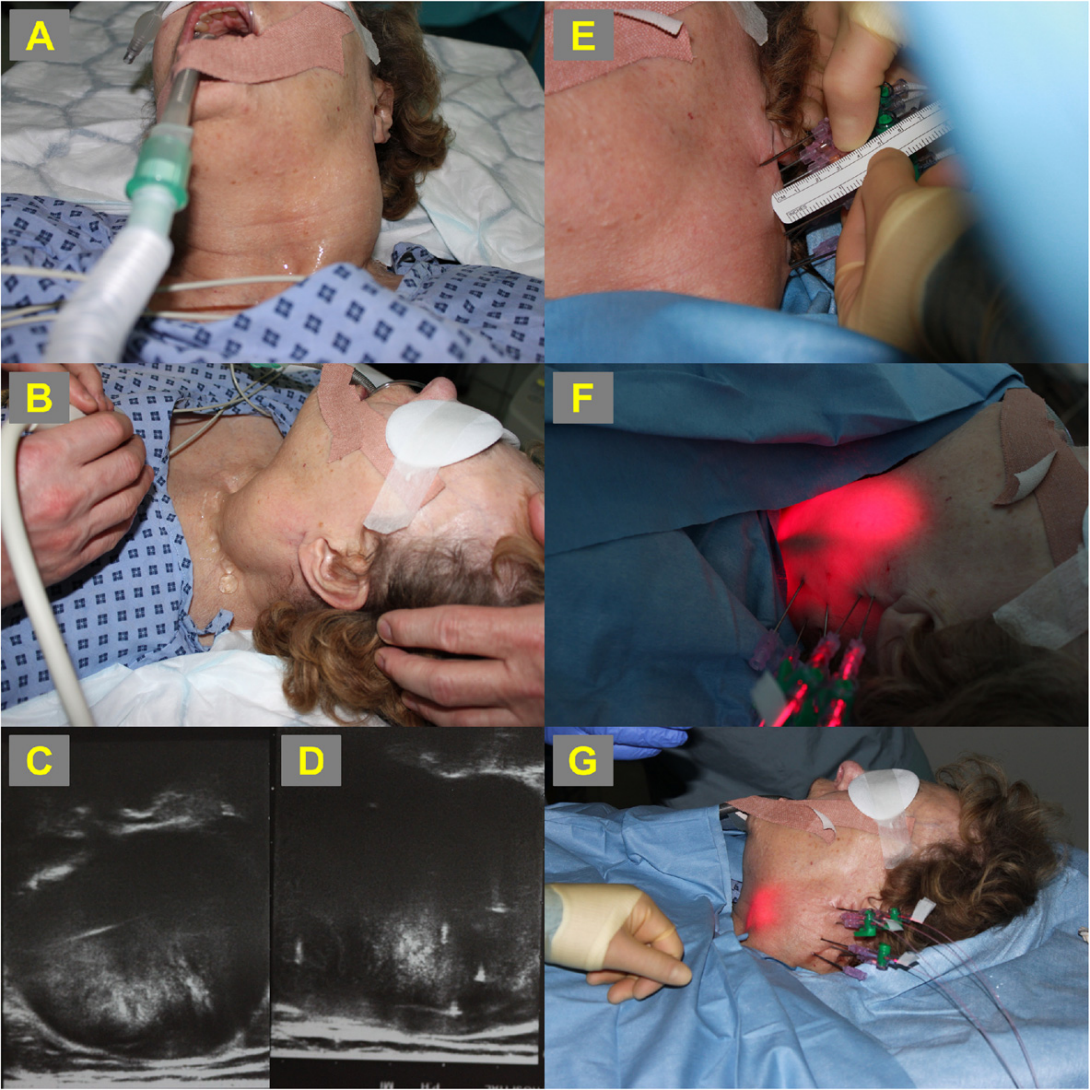
**PDT of neurofibroma. A**: Intra-operative image showing the large mass of the left neck. **B**: Intra-operative ultrasonography assessing the centre and periphery of the lesion as well as any neurovascular involvement or involvement of hollow organs. **C** and **D**: On-table US images showing the neurofibroma and its boundaries. **E**: Needle insertion into lesion prior to fibre insertion. **F** and **G**: light delivery to initiate the photochemical reaction.

Intra-operatively, an US scan of the area was performed to assess the centre and periphery of the lesion, as well as its involvement with any neurovascular structures or hollow organs. Spinal needles were then fed into the tumour under US-guidance. Optical fibres were then inserted through the needles and allowed to protrude by 2-3 mm to deliver maximum light to the abnormal tissue. As the diameter of necrosis post-PDT does not exceed 1 cm, the needles were pulled back 1 cm at a time to allow treatment of the whole tumour mass. The total energy delivered per port was 20 J/cm^2^ (Figure 1).

Pain, with slight dysphagia and odynophagia were the main issue in the immediate post-PDT phase. The pain was manageable by simple analgesics and settled completely within 5–7 days.

Post-PDT follow-up showed that the patient’s pain, dysphagia and shortness of breath issues had been improved. The disfigurement of the neck caused by the mass was no longer evident. Three months post-PDT, MRI revealed a significant reduction in the neurofibroma size (Figure 1). The patient is now four years post-intervention and continues to complain of no quality of life issues.

**Figure 2:**
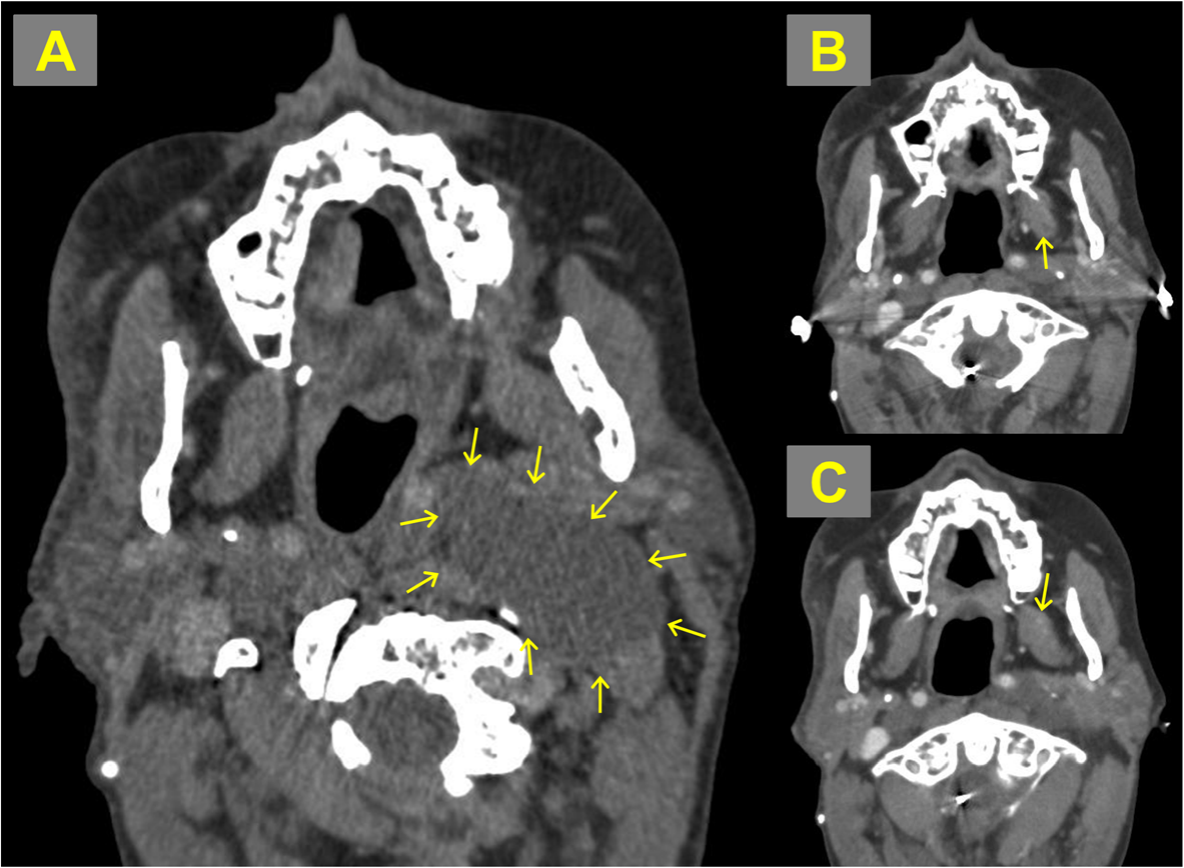
**Axial MRI scanning of neurofibroma of the neck. A**: Baseline MRI showing a large lesion of the left neck compressing the nearby hollow organs, causing a slight breathing and swallowing compromise. **B** and **C**: three months post-PDT scans, slightly upper and lower to the pre-PDT scan level, showing significant shrinkage with an increase in airway patency.

## Discussion

The gold-standard treatment for neurofibromas continues to be surgery. In the head and neck, this often presents a challenge because complete excision of these benign tumours may result in greater morbidity due to the complicated anatomy. In some cases, complete excision may require sacrifice of the cranial nerves, causing significant functional and loco-regional deficits, or result in substantial cosmetic deformity. Subtotal resection may allow reduced morbidity but inevitably leads to recurrence [1-4].

There have been very few alternatives to surgery. Radiotherapy is often not feasible when dealing with a diffuse form of the disease. For solitary plexiform neurofibroma of the head and neck, however, radiotherapy may have a role in shrinking these lesions and controlling their growth. Intensity-modulated radiotherapy was shown to be effective in controlling extensive or recurrent juvenile angiofibroma [2].

Our patient had a lesion causing refractory pain, dysphagia and some breathing problems, especially on exertion. Although resection has been the treatment of choice for these lesions, PDT was employed due to the patient’s choice. PDT led to significant reduction in the lesion size after only one round of treatment. This has led to complete symptoms resolution with no morbidity. Successful treatment of these lesions does not require a complete PDT response and the possibility remains that a further round of PDT could be employed, especially in the case of regrowth and recurrence of symptoms.

We believe that the application of three-dimensional PDT can be instrumental in eliminating disease and reduces the chances of recurrence. The aim here was to ensure that the disease margins are dealt with and nothing gets missed. With regards to neurofibromas, due to the non-malignant nature of the disease, shrinkage and control of disease progression and symptoms may be satisfactory to the patient and also avoid exposing the patient to a major and sometimes disfiguring surgery.

Although the patient continues to be symptom-free after four years since intervention, continuous surveillance is recommended due to the small risk of malignant transformation. This has been reported to occur infrequently with acoustic neuromas treated with radiotherapy [3,4].

## Conclusion

Solitary plexiform neurofibromas of the head and neck occurring outside the setting of NF-I are rare. Because of the infiltrative quality of these lesions, complete surgical excision can be difficult and may result in significant morbidity. PDT has been used successfully to treat similar benign tumours of the head and neck. Current state-of-the-art PDT techniques, which can limit harmful effects to surrounding normal structures, provide a viable therapeutic option in cases where complete excision poses an unjustifiably high risk of morbidity. This case study showed that PDT was effective in shrinking the neurofibroma and controlling its progression leading to symptom control and no morbidity.

## Consent

Written informed consent was obtained from the patient for publication of this case study and accompanying images. A copy of the written consent is available for review by the Editor-in-Chief of this journal.

## Competing interests

The authors declare that they have no competing interests.

## Authors’ contributions

ZH, WJ, RA and CH contributed to conception and design, carried out the literature research, manuscript preparation and manuscript review. All authors have read and approved the final version of the manuscript.
